# An analysis of heart failure among cancer patients in the United States between 1999 and 2020

**DOI:** 10.3389/fonc.2026.1896502

**Published:** 2026-08-05

**Authors:** Abes A. Bautista Neughebauer, Deepak Talreja, Apostolos Gaitanidis

**Affiliations:** 1 Department of Cardiology, Eastern Virginia Medical School, Norfolk, VA, United States; 2 Department of Surgery, Eastern Virginia Medical School, Norfolk, VA, United States

**Keywords:** cancer, cardio-oncology, epidemiology, heart failure, oncology

## Abstract

**Background:**

Cancer and heart failure (HF) are highly prevalent in the population and can co-exist, due to shared risk factors and an aging population. The HF-related mortality burden in cancer patients across in the United States (US) remains poorly understood. The goal of this study is to evaluate the patterns of HF-related deaths among cancer patients in the United States.

**Methods:**

A retrospective analysis was performed using the Centers for Disease Control and Prevention (CDC) WONDER Multiple Cause of Death database between years 1999 and 2020. HF-related deaths were defined as death certificates listing HF as a contributing cause of death, while HF-specific deaths were defined as those with HF listed as the underlying cause of death. The ten most common malignancies in the US were analyzed. Mortality rates, temporal trends, regional differences, sex- and race-based disparities were evaluated.

**Results:**

A total of 402,308 HF-related deaths were identified among patients with the ten most common cancers. Lung cancer was associated with the highest rates, accounting for 115,283 deaths (1.71 per 100,000 population), followed by prostate cancer (1.08 per 100,000), colorectal cancer (0.90 per 100,000) and breast cancer (0.84 per 100,000). Most malignancies had increasing HF-related mortality during the latter years of the study period. The Midwest had the highest HF-related mortality rates for several types of cancer. White patients had the highest HF-related mortality rates across all cancer types. Among HF-specific deaths, prostate cancer exhibited the highest mortality rate (594.03 per 100,000).

**Conclusions:**

There is a substantial HF-related mortality burden across cancer patients, which is on the rise in recent years. Certain high-risk populations may be identified and cardio-oncology surveillance and targeted cardiovascular risk reduction strategies may be implemented among cancer survivors.

## 1 Introduction

While prior research on heart failure (HF) and cancer has typically seen the two as separate entities, more recent studies are focusing on their intertwining nature. From the cardiotoxic effects of cancer therapy, the burden of multi-visceral resection, vascular remodeling associated with radiation, HF is more prevalent in cancer patients than previously thought (1). The use of transthoracic echocardiography, cardiac magnetic resonance imaging and multigated acquisition scans has made specialized HF assessment more prevalent and cancer patients can undergo detailed screening during all phases of their treatment. Patients receiving cardiotoxic cancer therapies are regularly evaluated for signs of heart failure through repeat echocardiograms, bloodwork and symptom assessment. There are various prediction models that have been developed to help stratify risk of such patients, though these are not standard of care at this time given limited external validation (1). Despite treatment effects, there are other mechanisms linking the two, such as circulating factors, specifically SerpinA3 and the gut microbiome (2, 3). Multiple risk factors are also common to both heart failure and cancer, including hypertension, diabetes and obesity. In the Framingham Heart Study and the Prevention of Renal and Vascular End-Stage Disease study, obesity and abdominal adiposity are associated with higher risks of specific cancers, underlying the presence of common underlying risk factors between the two (4).

Despite the vast literature describing the development of HF in the setting of such therapies, there are not many population studies illustrating the prevalence of HF among cancer patients. As such, the relationship between HF and cancer still remains inadequately understood. Given the advances in the field of HF in the past decade, the new understanding of its pathophysiology and the emerging role of the goal-directed medical therapy, it is important to better understand the relationship between HF and cancer. Also, the emergence of new therapies for several types of advanced cancers have made advanced cancer a long-term rather than short-term problem and as a result, the prevention and treatment of underlying medical conditions, such as HF, is more important than ever. For example, there are numerous trials studying the benefit of starting preventative statin therapy, beta blockers and even neurohormonal antagonists as primary prevention of cardiac dysfunction in patients receiving high risk cancer treatment. There is also a much-needed effort to address comorbidities/risk factors linked to both HF and cancer, such as hypertension, tobacco use and type 2 diabetes. Cancer diagnosis is a reason for exclusion from HF clinical trials making the study of this patient population complicated. HF diagnosis is also used as a reason for exclusion from novel cancer therapy clinical trials, further underlying the importance of better understanding this patient population. In this study, we aim to better understand the HF-related mortality burden of HF among cancer decedents in the United States between 1999 and 2020.

## 2 Methods

Data was obtained from the Centers for Disease Control and Prevention (CDC) WONDER Multiple Cause of Death (MCOD) database for years 1999–2020 (https://wonder.cdc.gov/). The MCOD database is extracted from death certificates filed across all 50 US states and the District of Columbia. It contains information pertaining to the underlying cause of death and contributing causes of death and the International Classification of Diseases, Tenth Revision (ICD-10) is used to identify specific diagnoses. Additional demographic and geographic variables are provided, as well as age-adjusted mortality estimates based on the US Census Bureau data.

A retrospective population-based study was conducted to assess the association between heart failure (HF) and the ten most common malignancies in the US. The following cancer types were considered based on relevant ICD-10 codes: Breast cancer (C50), Prostate cancer (C61), Lung cancer (C34), Colorectal cancer (C18–C20), Melanoma (C43), Bladder cancer (C67), Kidney cancer (C64–C65), Non-Hodgkin lymphoma (NHL, C82–C85), Endometrial cancer (C54–C55) and Pancreatic cancer (C25). The diagnosis of heart failure was determined using the ICD-10 code I50. Due to the use of deidentified patient information and publicly available data, Institutional Review Board approval was waived.

-As *HF-related mortality*, we defined mortality events where HF was a contributing underlying diagnosis listed as MCOD and the cancer of interest was listed anywhere on the death certificate as a multiple/contributing cause.
-As *HF-specific mortality*, we defined mortality events where HF was listed as the primary underlying cause of death and the cancer of interest was listed anywhere on the death certificate as a multiple/contributing cause

### 2.1 HF-related deaths among cancer decedents

The first analysis focused on HF-related deaths among decedents with each of the ten cancer types with a goal to estimate the burden of HF-related mortality with each cancer diagnosis. For each cancer type, death certificates containing both the specific cancer and the diagnosis of HF as MCOD were considered. The number of HF-related deaths fulfilling these criteria was determined and then mortality rates per 100,000 people were calculated using standard CDC Wonder methodology.

The results for each cancer type were stratified by: US Census region, US State, year of death (1999–2020), sex and race/ethnicity. Sex, race and ethnicity were defined based on CDC Wonder classifications.

### 2.2 HF-specific deaths among cancer decedents

HF-specific mortality was then examined for each cancer type. For this analysis, HF was specified as the underlying cause of death and each cancer type as a contributing cause of death. HF-specific mortality rates per 100,000 people were determined as the ratio of all deaths with HF as the underlying cause of death over the number of deaths associated with this specific cancer type that were attributed to other underlying causes of death.

For each cancer type, proportionate mortality ratios (PMRs) were calculated based on CDC guidance for death-certificate analyses (5), by dividing the observed number of HF deaths among decedents with each cancer by the expected proportion of HF deaths among all deaths in the general population. The numerator used for the calculation was the proportion of HF-specific deaths divided by the number of deaths from all causes among patients with each cancer type. The denominator used was the proportion of HF-specific deaths divided by the number of deaths from all causes in the US population per CDC instructions (5). These were meant to assess the relative contribution of HF-specific mortality among all cancer-associated deaths compared with the general distribution of HF-related mortality in the general population.

### 2.3 State-level analyses

Analyses at the state-level were performed to estimate the burden of HF-related mortality for the ten most common types of cancer. For this analysis, HF-related deaths were aggregated for all ten cancer types. For each state, the age-adjusted rate of HF listed as a contributing cause of death was determined. Additionally, the age-adjusted rate of HF listed as the underlying cause of death was determined. State-level maps showing those two metrics were made.

### 2.4 Statistical analysis

Analysis was performed using CDC Wonder and 95% confidence intervals were used. State-level maps were created using CDC Wonder. Other figures and text editing were performed using ChatGPT v5.5 (OpenAI, CA USA).

## 3 Results

A total of 400,982 HF-related deaths were identified among the top ten most common cancers in the US between 1999 and 2020.

### 3.1 HF-related mortality

Lung cancer demonstrated the highest burden of HF-related deaths, with 115,283 deaths and a crude mortality rate of 1.71 per 100,000 population (95% CI 1.70–1.72). Prostate cancer (73,155 deaths, 1.08 per 100,000), colorectal cancer (60,922 deaths, 0.90 per 100,000) and breast cancer (56,806 deaths, 0.84 per 100,000) also demonstrated substantial burden. Conversely, melanoma (6,040 deaths, 0.09 per 100,000) and endometrial cancer (7,968 deaths, 0.12 per 100,000) had the lowest rates. Detailed information is presented in **Table 1**.

**Table 1:** HF-related mortality by cancer type.

Cancer Type	Deaths	Crude rate per 100,000 (95% CI)
Breast cancer	56,806	0.84 (0.84 - 0.85)
Prostate cancer	73,155	1.08 (1.08 - 1.09)
Lung cancer	115,283	1.71 (1.70 - 1.72)
Colorectal cancer	60,922	0.90 (0.90 - 0.91)
Melanoma	6,040	0.09 (0.09 - 0.09)
Bladder cancer	25,580	0.38 (0.37 - 0.38)
Renal cell carcinoma	16,945	0.25(0.25 - 0.25)
Non-Hodgkin’s Lymphoma	34,736	0.51 (0.51 - 0.52)
Endometrial cancer	7,968	0.12 (0.12 - 0.12)
Pancreatic cancer	16,482	0.24 (0.24 - 0.25)

### 3.2 Temporal trends

**Figure 1:**
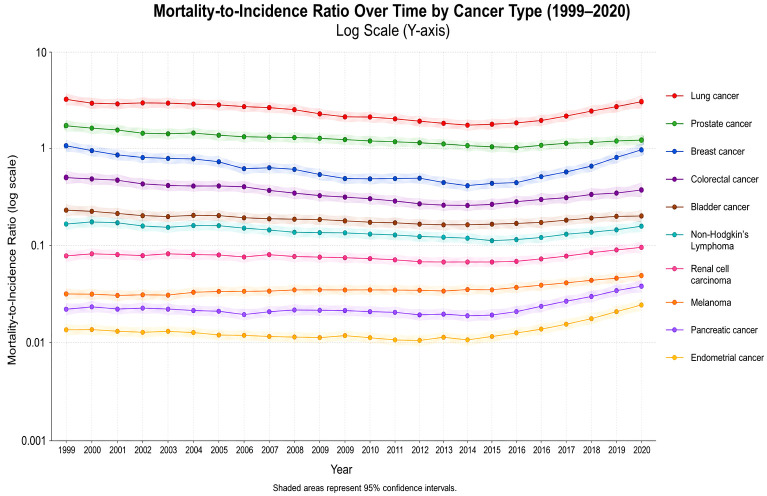
Temporal changes in heart-failure related mortality over time.

Lung cancer consistently demonstrated the highest HF-related mortality rates throughout the study period, with rates ranging from 1.82 in 1999 to 2.13 per 100,000 in 2020. Breast cancer demonstrated a decline in HF-related mortality from 1.02 per 100,000 in 1999 to a nadir of 0.70 per 100,000 in 2012, followed by a gradual increase reaching 1.00 per 100,000 by 2020. Most cancer types similarly demonstrated progressive increases in HF-related mortality towards the end of the study period. Detailed information is shown in **Table 2**. Trends over time for all 10 cancer types listed are viewed in **Figure 1**.

**Table 2:** Change in HF-related mortality per 100,000 by year.

Cancer Type	1999	2000	2001	2002	2003	2004	2005	2006	2007	2008	2009	2010	2011	2012	2013	2014	2015	2016	2017	2018	2019	2020
Breast cancer	1.02 (0.98-1.06)	1 (0.96-1.03)	0.96 (0.92-0.99)	0.95 (0.91-0.99)	0.94 (0.91-0.98)	0.88 (0.84-0.91)	0.89 (0.86-0.93)	0.86 (0.82-0.89)	0.79 (0.76-0.82)	0.77 (0.74-0.8)	0.77 (0.74-0.8)	0.77 (0.74-0.8)	0.74 (0.71-0.77)	0.7 (0.67-0.73)	0.72 (0.69-0.74)	0.72 (0.69-0.75)	0.76 (0.73-0.79)	0.78 (0.75-0.81)	0.79 (0.76-0.82)	0.87 (0.83-0.9)	0.91 (0.88-0.94)	1 (0.97-1.04)
Prostate cancer	1.37 (1.33-1.42)	1.34 (1.3-1.38)	1.29 (1.25-1.33)	1.25 (1.21-1.3)	1.21 (1.17-1.25)	1.17 (1.13-1.21)	1.15 (1.11-1.19)	1.12 (1.08-1.16)	1.04 (1.01-1.08)	1.03 (1-1.07)	1 (0.97-1.04)	1 (0.97-1.04)	0.93 (0.9-0.97)	0.91 (0.88-0.94)	0.91 (0.88-0.95)	0.89 (0.86-0.93)	0.93 (0.89-0.96)	0.96 (0.92-0.99)	1 (0.96-1.03)	1.06 (1.02-1.09)	1.11 (1.07-1.14)	1.29 (1.25-1.33)
Lung cancer	1.82 (1.77-1.87)	1.78 (1.73-1.83)	1.78 (1.73-1.82)	1.79 (1.74-1.84)	1.78 (1.74-1.83)	1.77 (1.72-1.82)	1.76 (1.71-1.81)	1.66 (1.62-1.71)	1.57 (1.53-1.61)	1.55 (1.51-1.6)	1.58 (1.54-1.63)	1.58 (1.54-1.62)	1.51 (1.47-1.55)	1.5 (1.46-1.54)	1.52 (1.48-1.56)	1.57 (1.52-1.61)	1.62 (1.58-1.66)	1.65 (1.6-1.69)	1.72 (1.68-1.77)	1.9 (1.85-1.95)	2.03 (1.98-2.08)	2.13 (2.08-2.18)
Colorectal cancer	1.22 (1.18-1.26)	1.21 (1.17-1.25)	1.13 (1.1-1.17)	1.11 (1.07-1.15)	1.14 (1.1-1.18)	1.06 (1.02-1.09)	1.03 (0.99-1.07)	0.98 (0.95-1.02)	0.91 (0.88-0.95)	0.87 (0.84-0.9)	0.81 (0.78-0.84)	0.81 (0.78-0.84)	0.77 (0.74-0.8)	0.75 (0.72-0.78)	0.72 (0.69-0.75)	0.74 (0.71-0.77)	0.71 (0.68-0.73)	0.74 (0.71-0.77)	0.74 (0.71-0.77)	0.82 (0.79-0.85)	0.84 (0.8-0.87)	0.93 (0.89-0.96)
Melanoma	0.07 (0.06-0.08)	0.07 (0.06-0.08)	0.08 (0.07-0.09)	0.07 (0.06-0.08)	0.07 (0.06-0.08)	0.07 (0.06-0.08)	0.08 (0.07-0.09)	0.08 (0.07-0.09)	0.08 (0.07-0.09)	0.08 (0.07-0.09)	0.07 (0.07-0.08)	0.08 (0.07-0.09)	0.08 (0.07-0.09)	0.08 (0.07-0.09)	0.1 (0.09-0.11)	0.1 (0.08-0.11)	0.1 (0.09-0.11)	0.1 (0.09-0.11)	0.11 (0.1-0.12)	0.13 (0.11-0.14)	0.12 (0.11-0.14)	0.14 (0.13-0.16)
Bladder cancer	0.37 (0.35-0.4)	0.37 (0.35-0.39)	0.36 (0.33-0.38)	0.36 (0.34-0.38)	0.34 (0.32-0.37)	0.36 (0.34-0.38)	0.37 (0.35-0.4)	0.36 (0.34-0.38)	0.36 (0.34-0.38)	0.34 (0.32-0.36)	0.33 (0.31-0.35)	0.34 (0.32-0.36)	0.33 (0.31-0.35)	0.36 (0.34-0.38)	0.35 (0.33-0.37)	0.36 (0.34-0.38)	0.38 (0.36-0.41)	0.4 (0.37-0.42)	0.41 (0.39-0.43)	0.44 (0.42-0.46)	0.49 (0.47-0.52)	0.52 (0.5-0.55)
Renal cell carcinoma	0.21 (0.19-0.23)	0.24 (0.22-0.25)	0.23 (0.21-0.24)	0.22 (0.21-0.24)	0.21 (0.2-0.23)	0.25 (0.23-0.27)	0.23 (0.21-0.24)	0.23 (0.22-0.25)	0.23 (0.21-0.25)	0.22 (0.21-0.24)	0.23 (0.21-0.24)	0.23 (0.21-0.24)	0.23 (0.21-0.24)	0.24 (0.22-0.26)	0.25 (0.23-0.26)	0.25 (0.24-0.27)	0.26 (0.25-0.28)	0.28 (0.26-0.3)	0.28 (0.26-0.3)	0.31 (0.29-0.33)	0.33 (0.31-0.35)	0.35 (0.33-0.37)
Non-Hodgkin’s Lymphoma	0.53 (0.5-0.56)	0.55 (0.52-0.57)	0.51 (0.49-0.54)	0.52 (0.5-0.55)	0.53 (0.5-0.55)	0.52 (0.49-0.55)	0.52 (0.49-0.54)	0.52 (0.49-0.54)	0.48 (0.46-0.51)	0.48 (0.45-0.5)	0.47 (0.45-0.49)	0.47 (0.45-0.5)	0.46 (0.44-0.48)	0.46 (0.43-0.48)	0.44 (0.41-0.46)	0.46 (0.44-0.48)	0.49 (0.47-0.51)	0.52 (0.5-0.55)	0.53 (0.5-0.55)	0.58 (0.55-0.6)	0.62 (0.59-0.64)	0.67 (0.65-0.7)
Endometrial cancer	0.13 (0.12 - 0.15)	0.14 (0.13 - 0.15)	0.13 (0.12 - 0.15)	0.12 (0.11 - 0.13)	0.12 (0.10 - 0.13)	0.11 (0.10 - 0.13)	0.13 (0.11 - 0.14)	0.11 (0.10 - 0.12)	0.11 (0.10 - 0.12)	0.1 (0.09 - 0.12)	0.09 (0.08 - 0.11)	0.11 (0.10 - 0.12)	0.09 (0.08 - 0.10)	0.1 (0.09 - 0.11)	0.09 (0.08 - 0.10)	0.1 (0.09 - 0.11)	0.11 (0.10 - 0.12)	0.12 (0.11 - 0.13)	0.12 (0.11 - 0.13)	0.13 (0.12 - 0.14)	0.15 (0.14 - 0.16)	0.17 (0.16 - 0.19)
Pancreatic cancer	0.24 (0.22-0.26)	0.23 (0.21-0.25)	0.23 (0.21-0.24)	0.24 (0.22-0.26)	0.21 (0.2-0.23)	0.21 (0.19-0.23)	0.22 (0.2-0.24)	0.22 (0.2-0.24)	0.22 (0.2-0.23)	0.22 (0.2-0.24)	0.21 (0.19-0.22)	0.2 (0.18-0.21)	0.22 (0.2-0.23)	0.22 (0.2-0.23)	0.22 (0.2-0.23)	0.23 (0.22-0.25)	0.27 (0.25-0.28)	0.25 (0.23-0.27)	0.29 (0.27-0.31)	0.3 (0.29-0.32)	0.34 (0.32-0.36)	0.38 (0.35-0.4)

### 3.3 Regional trends

Across the different US Census regions, the Midwest consistently had the highest rate of HF-related mortality across several cancer types, including lung cancer (2.01 per 100,000), prostate cancer (1.29 per 100,000), colorectal cancer (1.13 per 100,000), bladder cancer (0.47 per 100,000) and NHL (0.64 per 100,000, **Table 3**).

**Table 3:** Variation in HF-related mortality by region.

Cancer Type	Northeast	Midwest	South	West
Breast cancer	0.93 (0.91 - 0.94)	1.04 (1.03 - 1.06)	0.74 (0.73 - 0.75)	0.74 (0.73 - 0.76)
Prostate cancer	1.11 (1.09 - 1.12)	1.29 (1.27 - 1.30)	0.95 (0.93 - 0.96)	1.10 (1.09 - 1.12)
Lung cancer	1.67 (1.65 - 1.69)	2.01 (1.99 - 2.03)	1.76 (1.74 - 1.78)	1.38 (1.36 - 1.39)
Colorectal cancer	1.01 (0.99 - 1.03)	1.13 (1.11 - 1.15)	0.79 (0.78 - 0.80)	0.79 (0.77 - 0.80)
Melanoma	0.09 (0.09 - 0.10)	0.10 (0.09 - 0.10)	0.08 (0.08 - 0.09)	0.09 (0.09 - 0.10)
Bladder cancer	0.44 (0.43 - 0.45)	0.47 (0.46 - 0.48)	0.31 (0.30 - 0.32)	0.36 (0.35 - 0.37)
Renal cell carcinoma	0.24 (0.23 - 0.25)	0.30 (0.29 - 0.31)	0.24 (0.24 - 0.25)	0.23 (0.22 - 0.24)
Non-Hodgkin’s Lymphoma	0.55 (0.54 - 0.56)	0.64 (0.63 - 0.66)	0.44 (0.43 - 0.45)	0.49 (0.47 - 0.50)
Endometrial cancer	0.14 (0.14 - 0.15)	0.16 (0.15 - 0.16)	0.10 (0.09 - 0.10)	0.10 (0.09 - 0.10)
Pancreatic cancer	0.26 (0.25 - 0.27)	0.26 (0.26 - 0.27)	0.23 (0.22 - 0.23)	0.24 (0.23 - 0.25)

### 3.4 Sex-based trends

In general, males demonstrated higher HF-related mortality compared to females across most malignancies, with more pronounced differences found in bladder cancer (0.58 vs 0.19 per 100,000), melanoma (0.12 vs 0.06 per 100,000) and renal cell carcinoma (0.32 vs 0.19 per 100,000). Colorectal, pancreatic and breast cancers demonstrated similar rates between males and females. Detailed information is provided in **Table 4**.

**Table 4:** Variation in HF-related mortality by sex.

Cancer Type	Female		Male	
	Deaths	Rate per 100,000	Deaths	Rate per 100,000
Breast cancer	56,009	1.63 (1.62-1.65)	797	0.02 (0.02-0.03)
Prostate cancer	–	–	73,155	2.21 (2.19 - 2.22)
Lung cancer	48,388	1.41 (1.40 - 1.42)	66,895	2.02 (2.00 - 2.03)
Colorectal cancer	31,042	0.91 (0.90 - 0.92)	29,880	0.90 (0.89 - 0.91)
Melanoma	2,141	0.06 (0.06 - 0.07)	3,899	0.12 (0.11 - 0.12)
Bladder cancer	6,375	0.19 (0.18 - 0.19)	19,205	0.58 (0.57 - 0.59)
Renal cell carcinoma	6,479	0.19 (0.18 - 0.19)	10,466	0.32 (0.31 - 0.32)
Non-Hodgkin’s Lymphoma	16,004	0.47 (0.46 - 0.47)	18,732	0.56 (0.56 - 0.57)
Endometrial cancer	7,968	0.23 (0.23 - 0.24)	–	–
Pancreatic cancer	8,286	0.24 (0.24 - 0.25)	8,196	0.25 (0.24 - 0.25)

### 3.5 Race and ethnicity

White individuals had the highest rates of HF-related deaths among all groups. For instance, in lung cancer white individuals’ HF-related death rate was 1.90 per 100,000, compared with 1.18 among African Americans and 0.44 among Asian/Pacific Islanders. Melanoma had the most pronounced racial disparity, with HF-related mortality rates of 0.11 per 100,000 among white individuals compared with 0.01 per 100,000 among African American and Hispanic populations. Detailed characteristics are viewed in **Table 5**.

**Table 5:** Variation in HF-related mortality by race.

Cancer Type	White		African-American		Asian or Pacific Islander		American Indian or Alaska Native		Hispanic	
	Deaths	Rate per 100,000	Deaths	Rate per 100,000	Deaths	Rate per 100,000	Deaths	Rate per 100,000	Deaths	Rate per 100,000
Breast cancer	49,623	0.92 (0.92 - 0.93)	6,270	0.68 (0.67 - 0.70)	714	0.19 (0.18 - 0.21)	199	0.23 (0.19 - 0.26)	1,899	0.18 (0.17 - 0.18)
Prostate cancer	63,077	1.18 (1.17 - 1.18)	8,868	0.96 (0.94 - 0.99)	944	0.25 (0.24 - 0.27)	266	0.30 (0.26 - 0.34)	2,725	0.25 (0.24 - 0.26)
Lung cancer	102,212	1.90 (1.89 - 1.92)	10,853	1.18 (1.16 – 1.20)	1,637	0.44 (0.42 - 0.46)	581	0.66 (0.60 - 0.71)	3,118	0.29 (0.28 - 0.30)
Colorectal cancer	54,222	1.01 (1.00 - 1.02)	5,510	0.60 (0.58 - 0.62)	929	0.25 (0.23 - 0.27)	261	0.30 (0.26 - 0.33)	2,128	0.20 (0.19 - 0.21)
Melanoma	5,924	0.11 (0.11 - 0.11)	86	0.01 (0.01 - 0.01)	22	0.01 (0.00 - 0.01)	–	–	93	0.01 (0.01 - 0.01)
Bladder cancer	24,061	0.45 (0.44 - 0.45)	1,202	0.13 (0.12 - 0.14)	250	0.07 (0.06 - 0.08)	67	0.08 (0.06 - 0.10)	634	0.06 (0.05 - 0.06)
Renal cell carcinoma	15,055	0.28 (0.28 - 0.28)	1,544	0.17 (0.16 - 0.18)	224	0.06 (0.05 - 0.07)	122	0.14 (0.11 - 0.16)	900	0.08 (0.08 - 0.09)
Non-Hodgkin’s Lymphoma	32,096	0.60 (0.59 - 0.60)	1,946	0.21 (0.20 - 0.22)	573	0.15 (0.14 - 0.17)	121	0.14 (0.11 - 0.16)	1,519	0.14 (0.13 - 0.15)
Endometrial cancer	6,739	0.13 (0.12 - 0.13)	1,060	0.12 (0.11 - 0.12)	135	0.04 (0.03 - 0.04)	34	0.04 (0.03 - 0.05)	336	0.03 (0.03 - 0.03)
Pancreatic cancer	14,085	0.26 (0.26 - 0.27)	1,975	0.21 (0.21 - 0.22)	332	0.09 (0.08 - 0.10)	90	0.10 (0.08 - 0.13)	840	0.08 (0.07 - 0.08)

### 3.6 HF-specific deaths

In the overall US population, there were 1,427,576 deaths principally attributed to HF during the study period, corresponding to a rate of 21.16 per 100,000 people.

In terms of HF-specific mortality, prostate cancer had the highest rate at 594.03 per 100,000, followed by NHL (424.03 per 100,000), breast cancer (399.83 per 100,000) and bladder cancer (380.16 per 100,000). Pancreatic cancer had the lowest HF-specific mortality rate at 59.70 per 100,000. Lung cancer similarly demonstrated relatively low HF-specific mortality (106.21 per 100,000) compared with its high HF-related death burden.

### 3.7 Proportionate mortality ratios

PMRs were used to quantify the relative proportion of HF deaths among all causes of death for each cancer type, relative to the proportion of HF deaths in the general US population. Prostate cancer had the highest PMR among all cancers (PMR 28.07), followed by NHL (PMR 20.04), breast cancer (PMR 18.89) and bladder cancer (PMR 17.97). Pancreatic cancer (PMR 2.82) and lung cancer (PMR 5.02) demonstrated relatively lower PMRs. Detailed information is viewed in **Table 6**.

**Table 6:** HF-specific mortality in cancer decedents.

Cancer Type	Deaths from HF	All individuals	HF-specific mortality rate per 100,000	PMR (O/E)
HF deaths in general population	1,427,576	6,746,356,647	21.16	-
Breast cancer	4,502	1,125,980	399.83	18.89
Prostate cancer	5,496	925,211	594.03	28.07
Lung cancer	3,823	3,599,577	106.21	5.02
Colorectal cancer	3,928	1,365,226	287.72	13.60
Melanoma	382	207,204	184.36	8.71
Bladder cancer	1,573	413,769	380.16	17.97
Renal cell carcinoma	952	339,484	280.43	13.25
Non-Hodgkin’s Lymphoma	2,381	561,521	424.03	20.04
Endometrial cancer	446	218,130	204.5	9.64
Pancreatic cancer	506	847,589	59.70	2.82

### 3.8 State-level variations

**Figure 2:**
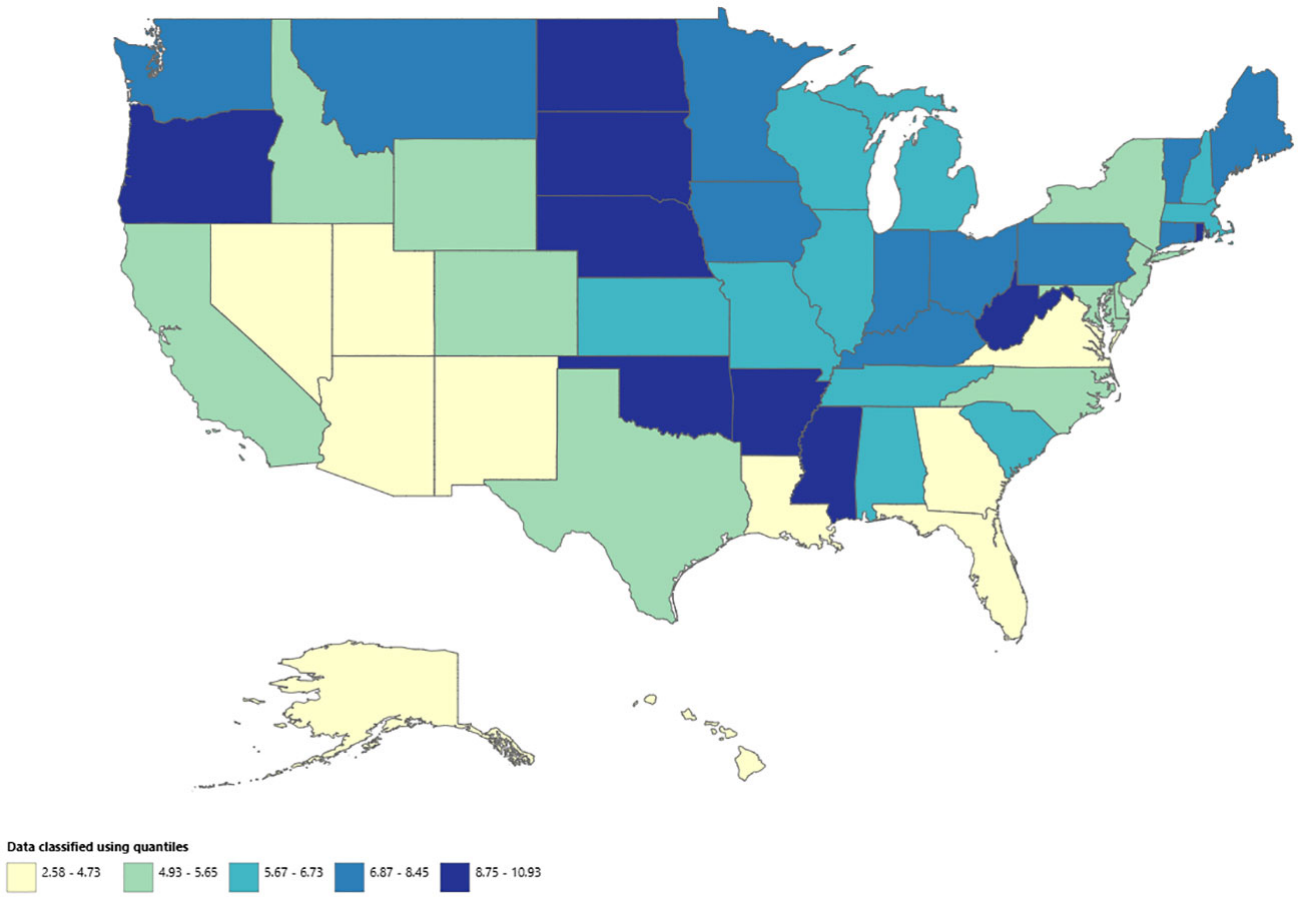
State-level representation of heart-failure related mortality among the ten most common cancers in the United States.

**Figure 3:**
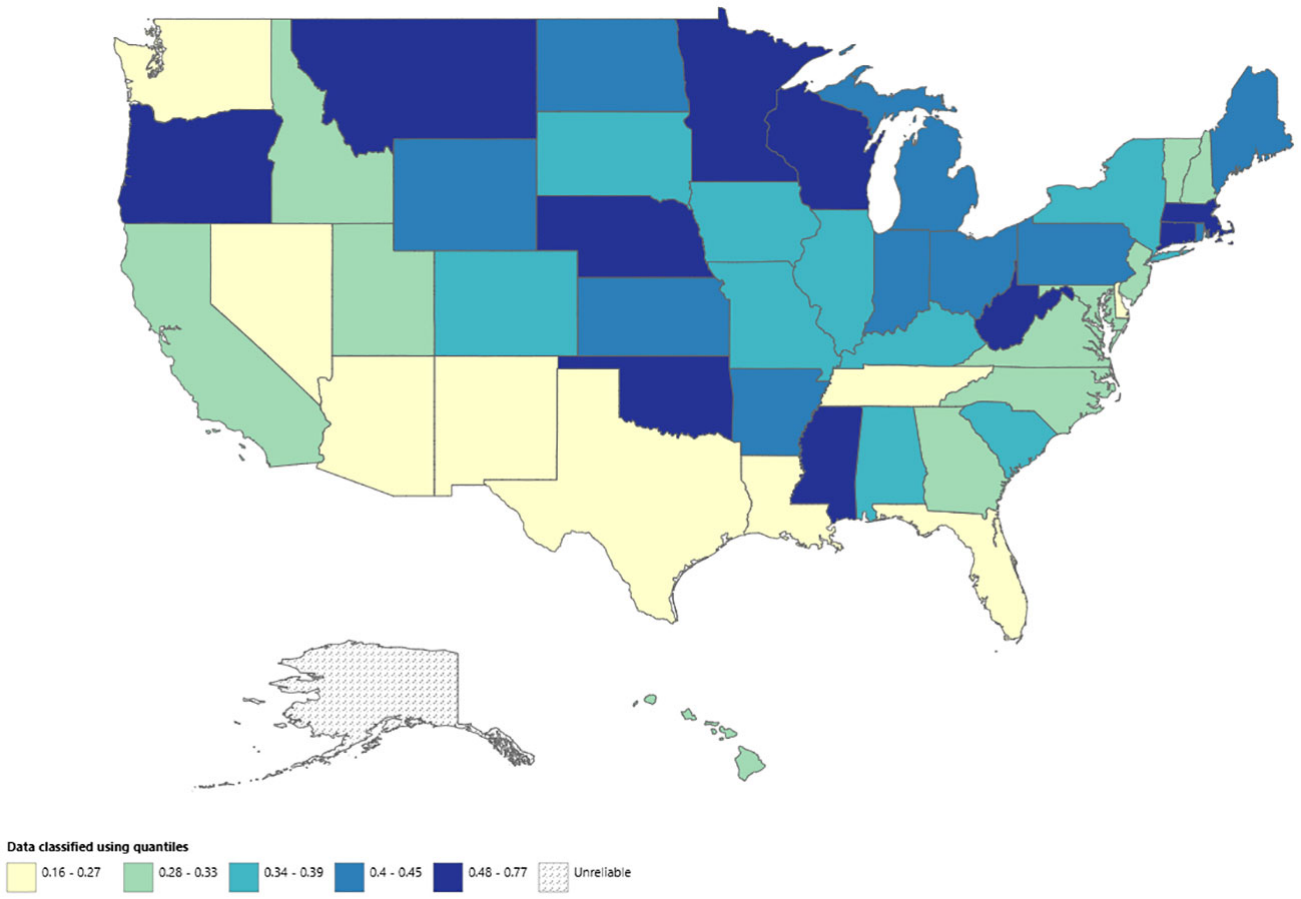
State-level representation of heart-failure specific mortality among the ten most common cancers in the United States.

State-level variation was observed in both HF-related mortality and HF-specific mortality. States with the highest HF-related mortality rates included North Dakota (10.93 per 100,000), West Virginia (9.68 per 100,000), Nebraska (9.70 per 100,000), South Dakota (9.57 per 100,000) and Mississippi (9.38 per 100,000). The highest HF-specific mortality rates were observed in Mississippi (0.77 per 100,000), Nebraska (0.73 per 100,000), Montana (0.63 per 100,000) and Oregon (0.59 per 100,000). State-level geographic distributions are shown in **Table 7**, **Figures 2** and **3**.

**Table 7:** State-level variation.

State	HF-Related Mortality Rate per 100,000	HF-Specific Mortality Rate per 100,000
Alabama	5.7	0.36
Alaska	2.93	Unreliable
Arizona	3.12	0.19
Arkansas	8.75	0.45
California	5.53	0.31
Colorado	5.07	0.35
Connecticut	6.89	0.52
Delaware	5.15	0.26
District of Columbia	4.08	0.18
Florida	4.53	0.16
Georgia	3.59	0.3
Hawaii	4.37	0.31
Idaho	5.65	0.33
Illinois	5.67	0.37
Indiana	7.07	0.43
Iowa	8.26	0.34
Kansas	6.73	0.41
Kentucky	7.95	0.38
Louisiana	4.73	0.2
Maine	7.27	0.4
Maryland	4.93	0.3
Massachusetts	5.82	0.49
Michigan	6.61	0.41
Minnesota	8	0.48
Mississippi	9.38	0.77
Missouri	6.48	0.39
Montana	6.95	0.63
Nebraska	9.7	0.73
Nevada	2.58	0.17
New Hampshire	6.47	0.33
New Jersey	5.48	0.32
New Mexico	4.26	0.23
New York	5.24	0.37
North Carolina	5.37	0.33
North Dakota	10.93	0.43
Ohio	8.45	0.44
Oklahoma	8.97	0.55
Oregon	9.02	0.59
Pennsylvania	7.85	0.43
Rhode Island	8.9	0.42
South Carolina	6.14	0.34
South Dakota	9.57	0.39
Tennessee	6.3	0.19
Texas	5.13	0.27
Utah	3.22	0.29
Vermont	8.45	0.28
Virginia	4.52	0.29
Washington	6.87	0.22
West Virginia	9.68	0.56
Wisconsin	6.6	0.5
Wyoming	4.95	0.44

## 4 Discussion

Among the ten most common malignancies in the United States, over 400 thousand HF-related deaths were identified between 1999-2020 of which over 110 thousand HF-related deaths were in the setting of lung cancer. Lung cancer consistently had the highest HF-related mortality among all cancers. For most malignancies, there was an increase in HF-related deaths towards the end of the study period, with the highest rates recorded by 2020. Men and white patients had the highest HF-related mortality rates. These findings highlight important trends in mortality linked to HF and, given the advances in the field of HF treatment over the past decade, highlight populations and cancer groups that may warrant further study and enhanced cardiovascular surveillance.

We found interesting variations amongst different geographic locations. The highest HF-related mortality rates spanned across multiple types of cancers in the Midwest (e.g. lung, prostate, colorectal, bladder and NHL). In contrast, the South and West tended to demonstrate lower HF-related mortality rates. Among specific states, the highest rates were seen in Mississippi, North Dakota, Oklahoma and Nebraska. In general, males had higher HF-related mortality rates compared to females. Nonetheless, several cancers showed similar rates, namely pancreatic, breast and colorectal. This is not surprising given the underlying risk factors for cardiovascular disease. Lam et al. have demonstrated that men are more predisposed to HF with reduced Ejection Fraction (HFrEF) given a greater burden of macrovascular coronary artery disease and higher risk for myocardial infarction (6). Male sex itself is an independent predictor for cardiovascular disease per the Framingham Heart Study (7). A metanalysis of approximately 94 studies illustrated that female HF patients have lower risks of all-cause mortality compared with males (8). Several modifiable risk factors are more common among males, such as smoking and hypertension (9). Another important factor is the anti-inflammatory and cardioprotective role that estrogen plays in premenopausal women (10).

Racial and ethnic disparities were also seen. White individuals experienced the highest HF-related mortality rates across the top ten cancers studied, with melanoma having the most pronounced differences. This observation has several underlying factors, including under-documentation of HF, survivorship bias and healthcare disparities contributing to reduced access to care. One of the major factors possibly contributing to this trend could be documentation bias and the underdiagnosis of HF in minority groups. HF could be documented less consistently on death certificates for minorities given not only more difficulty accessing health care, but also differences in autopsy rates and documentation practices (11, 12). Additionally, reduced access to care for individuals of non-white race may contribute to reduced access to diagnostic modalities and specialist care, causing a falsely low incidence of HF. Among individuals with HF without cancer, Non-Hispanic Blacks are found to have the highest HF mortality rates (13, 14). Another important confounder is survivorship bias. White patients tend to have better cancer-specific survival and perhaps live long enough for heart failure to become the primary cause of death (15). On the other hand, competing causes of death and differences in cancer survival may influence whether HF is documented as a contributor to death and this may be more common among non-white patients.

When HF was listed as the underlying cause of death, termed HF-specific mortality, our findings were equally interesting. The highest HF-specific mortality rates were recorded for prostate, NHL, breast and bladder cancers, respectively. Pancreatic cancer had the lowest HF-specific mortality rate. Interestingly, although the highest overall HF-related death burden was seen with lung cancer, the HF-specific mortality rate was on the lower side. By analyzing PMRs, we assessed whether HF represented a disproportionate share of deaths involving each cancer type relative to the overall US population. Prostate cancer had the highest PMR, while pancreatic and lung cancers had the lowest. This may reflect the higher competing cancer-related mortality associated with pancreatic and lung cancers prior to HF becoming the main etiology of mortality, while patients with prostate cancer live longer and causes of death other than the underlying cancer predominate.

The high HF rates observed in lung cancer likely represent the multitude of risk factors pulmonary malignancy shares with heart failure, such as tobacco (16, 17). The American Heart Association underscores smoking as the “strongest link” with HF (9). Lung cancer patients have been found to have the highest prevalence of pre-existing cardiovascular disease (18, 19). And patients with cardiovascular disease have a 67% increased risk of lung cancer (9, 20, 21). Apart from this, the therapies used for lung cancer further contribute to HF development, such as thoracic radiation, platinum-based chemotherapy, VEGF inhibitors and immune checkpoint inhibitors (22–26), although treatment exposure could not be assessed in this dataset. Advanced lung cancer and lung resections can also increase cardiopulmonary stress and predispose to HF in selected patients through pulmonary vasculature remodeling and increased afterload of the right ventricle. Surprisingly, despite having the highest HF-related mortality, lung cancer had a lower HF-specific mortality rate likely reflecting the high mortality associated with lung cancer itself.

There are several limitations of the paper that need to be addressed. First, given that the CDC Wonder Multiple Causes of Death databases uses ICD-10 codes recorded on death certificates, this inherently introduces potential misclassification/coding inaccuracies. Therefore, the specific severity, subtype and cause of the individual’s heart failure was not able to be extrapolated. Additionally, specific details regarding the individual’s cancer were also not available, namely stage, treatment regimen/duration and exposure to cardiotoxic agents. Secondly, cardiovascular risk factors and comorbidities were not accounted for, which can lead to limited adjustment for confounding variables. Such examples include coronary artery disease, type 2 diabetes, tobacco use, baseline cardiac function and hypertension. Thirdly, HF-related deaths were defined as heart failure as one contributing cause of death, though not the ultimate cause. This may under or overestimate the actual burden of cardiovascular disease in cancer patients. Lastly, the disparities seen via sex, race, ethnicity and geographic analysis should be interpreted cautiously. The differences described may partially reflect the differences in various confounding variables, including healthcare access, socioeconomic factors and regional coding practices that were not completely defined in this database.

In summary, we described the burden of HF-related mortality among cancer decedents with the ten most common malignancies in the United States between 1999 and 2020. Common risk factors and cancer treatments increase the risk of HF in cancer patients, but these mechanisms could not be directly assessed using this dataset. The study is limited by the use of a large dataset where HF is diagnosed by death certificates and may not capture the true incidence of HF in cancer patients. Our findings underscore the importance of long-term cardiovascular surveillance in both cancer survivors. As cancer therapy continues to improve survival, the prevalence of HF and other cardiovascular comorbidities will become increasingly relevant. Early incorporation of comprehensive cardio-oncology care, risk stratification and multidisciplinary management and discussion may help develop strategies to reduce HF-related morbidity and mortality in such patients.

## Data Availability

Publicly available datasets were analyzed in this study. This data can be found here: https://wonder.cdc.gov/mcd-icd10.html.

## References

[1] Bloom MW Vo JB Rodgers JE Ferrari AM Nohria A Deswal A . Cardio-oncology and heart failure: a scientific statement from the Heart Failure Society of America. J Card Fail. (2025) 31:415–55. doi: 10.1016/J.CARDFAIL.2024.08.045 39419165 PMC12317758

[2] Meijers WC Maglione M Bakker SJL Oberhuber R Kieneker LM De Jong S . Heart failure stimulates tumor growth by circulating factors. Circulation. (2018) 138:678–91. doi: 10.1161/CIRCULATIONAHA.117.030816 29459363

[3] De Wit S Geerlings L Shi C Dronkers J Schouten EM Blancke G . Heart failure-induced microbial dysbiosis contributes to colonic tumour formation in mice. Cardiovasc Res. (2024) 120:612–22. doi: 10.1093/CVR/CVAE038 38400709 PMC11074794

[4] Liu EE Suthahar N Paniagua SM Wang D Lau ES Li SX . Association of cardiometabolic disease with cancer in the community. JACC CardioOncol. (2022) 4:69–81. doi: 10.1016/j.jaccao.2022.01.095 35492825 PMC9040108

[5] Centers for Disease Control and Prevention National Institute for Occupational Safety and Health . Steps required to calculate proportionate mortality ratios. In: Atlanta GA USA: CDC. (2021).

[6] Lam CSP Arnott C Beale AL Chandramouli C Hilfiker-Kleiner D Kaye DM . Sex differences in heart failure. Eur Heart J. (2019) 40:3859–68. doi: 10.1093/EURHEARTJ/EHZ835 31800034

[7] Murphy SP Ibrahim NE Januzzi JL . Heart failure with reduced ejection fraction: a review. JAMA. (2020) 324:488–504. doi: 10.1001/JAMA.2020.10262 32749493

[8] Qiu W Wang W Wu S Zhu Y Zheng H Feng Y . Sex differences in long-term heart failure prognosis: a comprehensive meta-analysis. Eur J Prev Cardiol. (2024) 31:2013–23. doi: 10.1093/EURJPC/ZWAE256 39101475

[9] Zhang L Iliescu C Ferencik M Finamore V Beavers C Patel AR . Coronary atherosclerosis in patients with cancer and survivors: a scientific statement from the American Heart Association. Circulation. (2026) 153:e916–33. doi: 10.1161/CIR.0000000000001391 41657216

[10] Mauvais-Jarvis F Bairey Merz N Barnes PJ Brinton RD Carrero JJ DeMeo DL . Sex and gender: modifiers of health, disease, and medicine. Lancet. (2020) 396:565–82. doi: 10.1016/S0140-6736(20)31561-0 32828189 PMC7440877

[11] Ahmed F Mirza TR Eltawansy S Khan Z Mashkoor Y Gohar N . Temporal and demographic disparities in mortality trends for heart failure and COPD-associated heart failure in U.S. adults: a 1999-2020 analysis of CDC WONDER data. Cardiovasc Pathol. (2025) 77:107735. doi: 10.1016/J.CARPATH.2025.107735 40154769

[12] Faizan M Singh P Haleem SM Zaman A Humayun MA Mubariz M . Demographics and mortality trends of hypertensive heart failure in the United States, 2006-2020: insights from the CDC WONDER database. J Int Med Res. (2026) 54. doi: 10.1177/03000605261417063 41956996 PMC13070146

[13] Fonarow GC Ahmad FS Ahmad T Albert NM Alexander KM Baker WL . HF STATS 2025: heart failure epidemiology and outcomes statistics an updated 2025 report from the Heart Failure Society of America. J Card Fail. (2026) 32:439–98. doi: 10.1016/J.CARDFAIL.2025.07.007 40987671

[14] Bozkurt B Ahmad T Alexander KM Baker WL Bosak K Breathett K . Heart failure epidemiology and outcomes statistics: a report of the Heart Failure Society of America. J Card Fail. (2023) 29:1412–51. doi: 10.1016/J.CARDFAIL.2023.07.006 37797885 PMC10864030

[15] Zhu C Shi T Jiang C Liu B Baldassarre LA Zarich S . Racial and ethnic disparities in all-cause and cardiovascular mortality among cancer patients in the U.S. JACC CardioOncol. (2023) 5:55–66. doi: 10.1016/j.jaccao.2022.10.013 36875907 PMC9982284

[16] Siegel RL Miller KD Wagle NS Jemal A . Cancer statistics, 2023. CA Cancer J Clin. (2023) 73:17–48. doi: 10.3322/CAAC.21763 36633525

[17] Ding N Shah AM Blaha MJ Chang PP Rosamond WD Matsushita K . Cigarette smoking, cessation, and risk of heart failure with preserved and reduced ejection fraction. J Am Coll Cardiol. (2022) 79:2298–305. doi: 10.1016/J.JACC.2022.03.377 35680180 PMC12711428

[18] El-Rayes M Nardi Agmon I Yu C Osataphan N Yu HA Hope A . Lung cancer and cardiovascular disease: common pathophysiology and treatment-emergent toxicity. JACC CardioOncol. (2025) 7:325–44. doi: 10.1016/j.jaccao.2025.05.003 40537184 PMC12228138

[19] Tarantini L Gallucci G Inno A Camerini A Canale ML Larocca M . A new era, new risks: the cardio-oncology perspective on immunotherapy in non-small cell lung cancer. Cancers (Basel). (2025) 17. doi: 10.3390/CANCERS17213443 41228236 PMC12609471

[20] Wang C Lu D Cronin-Fenton D Huang C Liew Z Wei D . Cardiovascular disease and risk of lung cancer incidence and mortality: a nationwide matched cohort study. Front Oncol. (2022) 12. doi: 10.3389/fonc.2022.950971 PMC9486704 36147909

[21] Wilcox NS Amit U Reibel JB Berlin E Howell K Ky B . Cardiovascular disease and cancer: shared risk factors and mechanisms. Nat Rev Cardiol. (2024) 21:617–31. doi: 10.1038/S41569-024-01017-X 38600368 PMC11324377

[22] Yegya-Raman N Berlin E Feigenberg SJ Ky B Sun L . Cardiovascular toxicity and risk mitigation with lung cancer treatment. Curr Oncol Rep. (2023) 25:433–44. doi: 10.1007/S11912-023-01387-4 36811807

[23] Kim Y Bates JE Yoon HI Grassberger C . Cardiac radiosensitivity in the era of thoracic chemoradiotherapy and immunotherapy: a scoping review. Lancet Oncol. (2026) 27:e130–40. doi: 10.1016/S1470-2045(25)00651-5 41785903 PMC13492064

[24] Mo Y Wei D Liang X Yan W Song Q Ma J . Treatment-related cardiotoxicity in non-small cell lung cancer: a population-based analysis for risk stratification. QJM. (2026). doi: 10.1093/QJMED/HCAG089 41883157

[25] Beavers CJ Rodgers JE Bagnola AJ Beckie TM Campia U Di Palo KE . Cardio-oncology drug interactions: a scientific statement from the American Heart Association. Circulation. (2022) 145:E811–38. doi: 10.1161/CIR.0000000000001056 35249373

[26] Du H Wang J Wang Z . Cardiovascular adverse effects of immunotherapy in cancer: insights and implications. Front Oncol. (2025) 15. doi: 10.3389/FONC.2025.1601808 40606990 PMC12213269

